# Assessment of educational needs of heart failure patients in intensive care units: qualitative study

**DOI:** 10.1186/2197-425X-3-S1-A208

**Published:** 2015-10-01

**Authors:** B Kiliç, H Sütçü Çiçek

**Affiliations:** Gulhane Military Medical Academy, School of Nursing, Internal Medicine Nursing Department, Ankara, Turkey

## Intr

Heart failure (HF) is a chronic and life-threatening disease which can be characterized with serious fatigue, dyspnea and deterioration of functional condition. Because of the life-threatening nature of the symptoms patients often apply to ER and referred to intensive care.

## Aim

The aim of this study is the assessment of educational needs of HF patients in intensive care units.

## Methods

A qualitative study to determine the experiences of the patients about their condition which affects the educational needs of the patients. The Data collected by individual in depth interview with 14 patients receiving treatment in the coroner intensive care unit are evaluated according to Colaizzi's phenomenological data analysis method.

## Findings

Average age of the patients in this study is 69.21(43-80) and %64.3 of them are females. Of the patients who have participated in the study %30.8 of them have stage III, %69.2 of them have stage IV HF. After their diagnosis, patients have been in intensive care for an average of 6.7 ± 4.4 (1-16) times. Average time period for the disease is 9.4 ± 7.5 years. In this study 3 categories for the educational needs of the patients and in each category 4 themes have been determined.

Category 1: Themes related to the educational needs of patients about usage of drugs,

(i) effects of drugs and, their purpose,
(ii) side effects of drugs,
(iii) drug-drug and drug-food interaction,
(iv) reasons for changing drugs.

Category 2: Themes related with lifestyle changes

(i) Nutritional characteristics and reasons for salt restriction,
(ii) coping with the symptoms of fatigue and weariness, planning the activities,
(iii) regulation of sleep
(iv) coping with stress.

Category 3: Themes about the educational needs of the patients related to the characteristics of the disease,

(i) prognosis of the disease, meaning and interpretation of the tests,
(ii) warning signs of the disease,
(iii) compliance with limitations,
(iv) control of the symptoms.

## Results

In this study HF patients stated that they mainly need information about the effects and purposes of the drugs they used. The need for information about the management of the symptoms that affect daily activities are considered 2^nd^ and the educational needs about the disease itself are considered 3^rd^ in importance.
